# The DNA-polymorphism rs849142 is associated with skin toxicity induced by targeted anti-EGFR therapy using cetuximab

**DOI:** 10.18632/oncotarget.25689

**Published:** 2018-07-13

**Authors:** Matthias F. Froelich, Sebastian Stintzing, Jörg Kumbrink, Thomas G.P. Grünewald, Ulrich Mansmann, Volker Heinemann, Thomas Kirchner, Andreas Jung

**Affiliations:** ^1^ Institute of Pathology, Medical Faculty, LMU Munich, Munich, Germany; ^2^ Department of Medicine III, University Hospital LMU Munich, Munich, Germany; ^3^ Comprehensive Cancer Center, University Hospital LMU Munich, Munich, Germany; ^4^ German Cancer Research Centre (DKFZ); German Cancer consortium (DKTK), Heidelberg, Germany; ^5^ Institute for Medical Informatics, Biometry, and Epidemiology, University of Munich, Munich, Germany; ^6^ Max-Eder Research Group for Pediatric Sarcoma Biology, Institute of Pathology, Medical Faculty, LMU Munich, Munich, Germany

**Keywords:** colorectal cancer, SNPs, skin toxicity

## Abstract

Skin toxicity (ST) is a frequent adverse effect (AE) in anti-epidermal growth factor receptor (EGFR)-targeted treatment of metastatic colorectal cancer (mCRC) resulting in decreased quality of life and problems in clinical management. We wanted to identify biomarkers predicting ST in this setting and focused on 70 DNA polymorphisms associated with acne, the (immunoglobulin fragment crystallizable region) Fcγ-receptor pathway, and systemic lupus erythematosus (SLE) applying next-generation-sequencing (NGS). For the analysis patients with mCRC treated with cetuximab were selected from the FIRE-3 study. A training group consisting of the phenotypes low (1) - and high-grade (3) ST (*n* = 16) and a validation group (*n* = 55) representing also the intermediate grade (2) were genotyped and investigated in a genotype-phenotype association analysis. The single nucleotide polymorphism (SNP) rs849142 significantly associated with ST in both the training- (*p* < 0.01) and validation-group (*p* = 0.04). rs849142 is located in an intron of the juxtaposed with another zinc finger protein 1 (*JAZF1*) gene. Haplotype analysis demonstrated significant linkage disequilibrium of rs849142 with *JAZF1*. Thus, rs849142 might be a predictive biomarker for ST in anti-EGFR treated mCRC patients. Its value in the clinical management of AE has to be validated in larger cohorts.

## INTRODUCTION

Skin toxicities (ST) like papulopustular rash (acneiform eruption), erythema, skin fissures, hair and nail changes, paronychia, and xerosis are frequent adverse effects (AE) when targeting the EGFR in solid cancers like metastatic colorectal cancer (mCRC), lung cancer, head and neck cancers and others [1]. ST occurs mostly at the face, scalp and trunk within the first three weeks or during the second cycle of treatment with variable severity in 82% of mCRC-patients receiving targeted anti-EGFR-treatment [2–5]. 38–70% of the patients also demonstrated bacterial but seldom viral infections at sites of ST [2, 6] all of which negatively affected quality of live [2, 7–9]. ST is treated pre-emptively and symptomatically applying special creams as well as oral and topical treatment [7, 10]. It is unknown if these compounds interfere with anti-EGFR targeted therapy. The severity of ST is classified by a simple three tier grading system -Common Toxicity Criteria for Adverse Events (CTCAE)-: grade 1 (mild; ~40% of the patients), grade 2 (moderate, ~40%), and grade 3 (severe, ~20%) (National Cancer Institute; CTCAE. http://evs.nci.nih.gov/) [3]. Interestingly, ST is an on-treatment marker for the efficacy of the anti-EGFR targeted therapy [2, 5, 8, 9, 11]. Despite its acneiform appearance, it differs both etiologically and pathologically from acne as for example comedones are missing [7]. Reasons for ST might be alterations in the cytokine/ chemokine profile, disturbed keratinocyte differentiation, barrier defects due to xerosis or infection [2]. Predictive biomarkers for ST were investigated and include a polymorphism in intron 1 of the EGFR-gene influencing expression levels [9, 12], expression of inflammatory lymphokines [13], and serum levels of EGFR-ligands [14–16]. Unfortunately, the predictive value of these biomarkers is either low or was not validated. Thus, none of these biomarkers found its way into clinical practice. Therefore, we searched for biomarkers which are predictive for ST in mCRC and identified the rs849142 SNP as a potentially predictive biomarker for ST in anti-EGFR treated mCRC patient cohort of the FIRE-3 study.

## RESULTS

### Skin toxicity is significantly associated with survival in a FIRE-3 study collective

**Figure 1:**
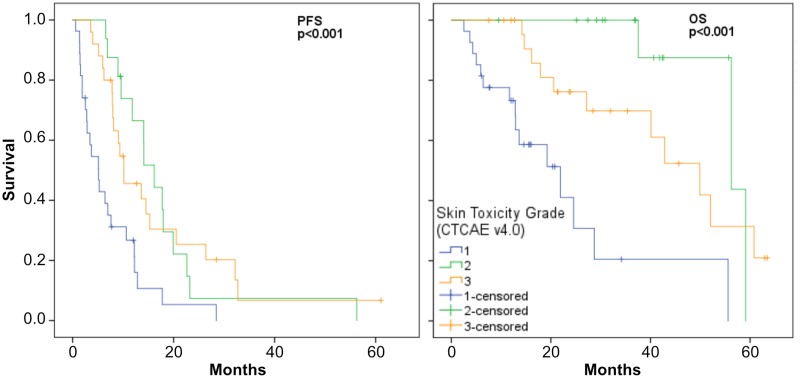
PFS and OS Kaplan–Meier plots for cetuximab associated ST grades

As only a limited number of specific biomarkers predicting ST is available which has not been approved for clinical use [16] our aim was to identify and validate potentially clinically useful predictive biomarkers for ST. Therefore, we chose a sub-collection of mCRC patients from the FOLFIRI plus cetuximab arm of the FIRE-3 study [17, 18]. A training/ validation approach was chosen including 16 randomly selected mCRC patients with ST grade 1 or 3 for the training group (mean age 64.2 years) and 55 patients in the validation group (mean age 62.4 years) with ST grade 1, 2, and 3 (Table 1). To see if this subpopulation of the FIRE-3 study still represented the complete group of patients we tested for the known correlation of the ST-grade induced by anti-EGFR therapy with the survival of the patients [9] employing Kaplan–Meier statistics. In the chosen subgroup ST grade and survival statistically correlated with both progression free survival (PFS; *p* < 0.001) and overall survival (OS; *p* < 0.001) comparably to the whole patient collective (Figure 1). Similar results were also obtained when the training- and validation sets were tested independently (PFS, *p*-training set = 0.016, *p*-validation set = 0.004; OS, *p*-training set = 0.059, *p*-validation set < 0.004) (Supplementary Figure 1).

**Table 1:** Composition of the evaluated training and validation patient groups

Group	Age	Sex			Skin toxicity		
	mean	male	female	unknown	1	2	3
**Training (*n* = 16)**	64.2	6 (37.50%)	7 (43.75%)	3 (18.75%)	11 (68.75%)	0 (0.00%)	5 (31.25%)
**Validation (*n* = 55)**	62.4	43 (78.18%)	12 (21.82%)	0 (0.00%)	19 (34.55%)	16 (29.09%)	20 (36.36%)

Thus, representative subgroups of patients of the clinical FIRE-3 study were available which were therefore enrolled as the data-basis for subsequent steps in the identification of predictive biomarkers for anti-EGFR therapy induced ST.

### SNPs known to be associated with acne, FcRγ, and SLE were selected for the investigation of ST in mCRC patients treated with cetuximab

**Figure 2:**
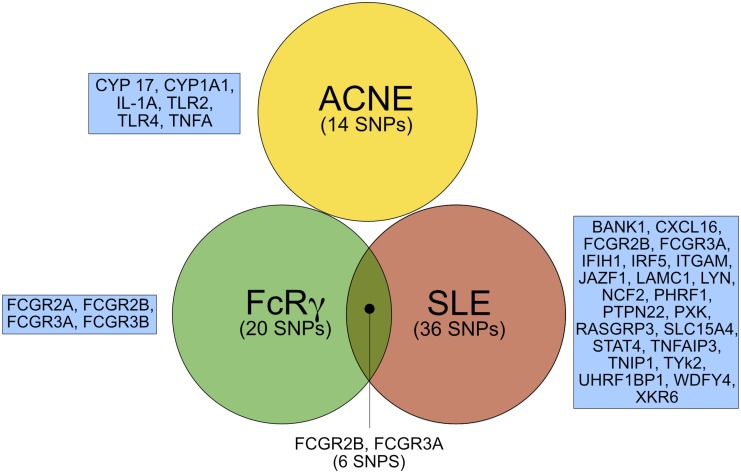
SNPs and respective genes selected for targeted sequencing (complete results in Supplementary Table 1) FcRγ, Fc receptor γ; SLE, systemic lupus erythematodes. SNP; single nucleotide polymorphism.

Because ST is most likely a tumor-independent AE mediated by anti-EGFR antibody treatment, we hypothesized that single nucleotide polymorphisms (SNP) which are known to be related to other ST like acne [19–21], systemic lupus erythematodes (SLE) [22], or FcRγ-signaling [23, 24] might also be indicative for anti-EGFR treatment related ST. Appropriate markers were selected from the literature [6, 19–24] and public databases (https://www.snpedia.com) (Figure 2). All together 14 SNP associated with acne, 20 SNPs with FcRγ, and 36 with SLE were identified as potential markers for ST (Figure 2). Six of these SNPs were found in both the SLE- as well as the FcRγ-subgroup. Thus, together 64 SNPs were employed in an NGS-approach for the investigation of ST in mCRC patients treated with anti-EGFR antibodies. After multiple steps of NGS quality control testing (Supplementary Figure 2) a set of 61 SNPs and 71 patients remained for the linkage analysis of SNPs associated with ST.

### SNP rs849142 is significantly associated with ST related to cetuximab

**Figure 3:**
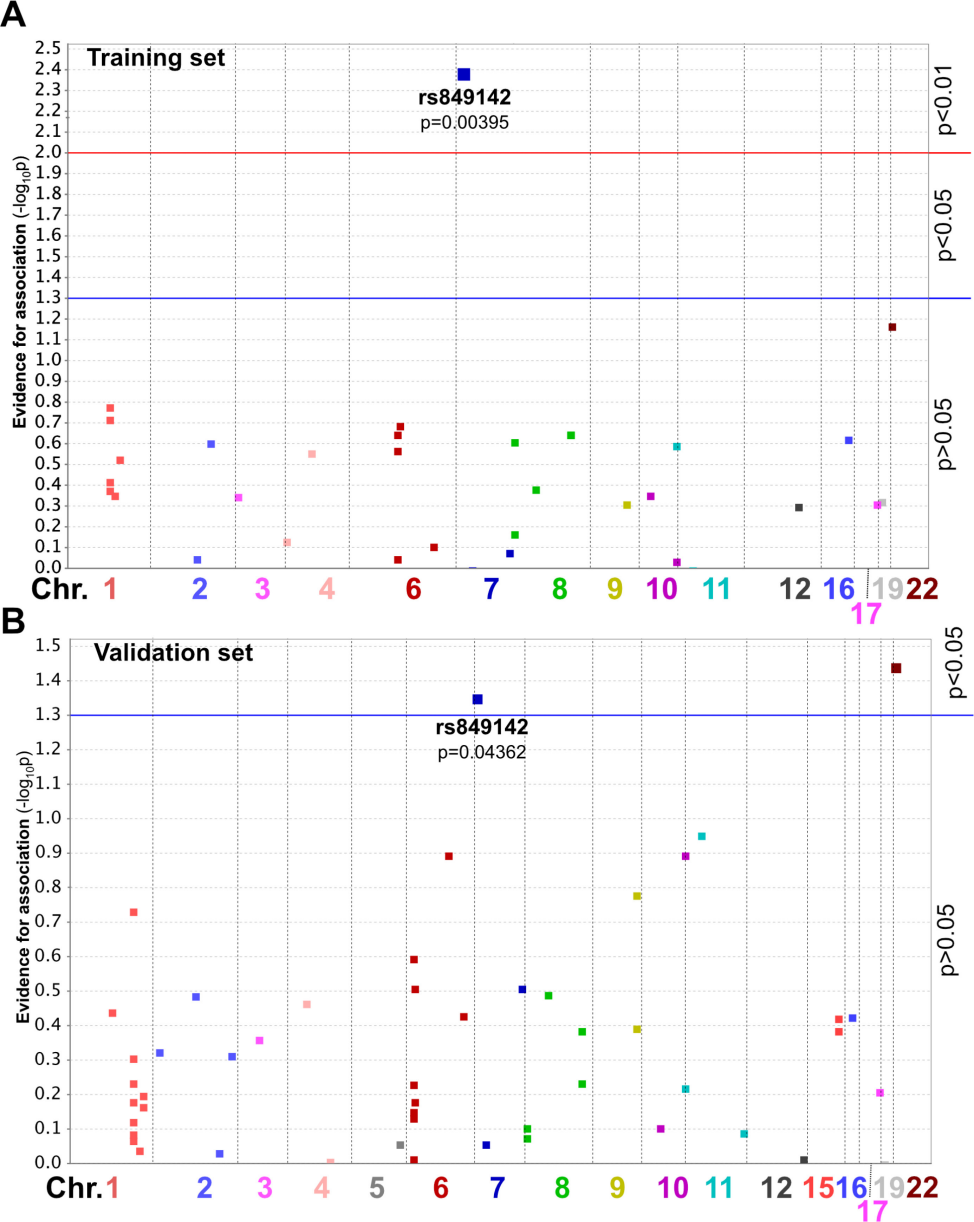
Manhattan plot for ST of patients from the Training group (**A**) or Validation group (**B**). SNP position on the chromosome is indicated by horizontal position. Y-axis value describes magnitude of evidence for association calculated as -log_10_(*p* value). Chr., chromosome.

To investigate a possible association between genotypes (SNPs) and severity of ST the genetic linkage analysis routine of the PLINK software [25] was applied for both the training- and validation set (Figure 3). It turned out that rs849142 (T allele) was significantly associated with ST in both the training- (*p* = 0.004, Sens = 100.00%, Spec = 83.33%, PPV = 80.00%, NPV = 100.00%, Figure 3A) and validation group (*p* = 0.0436, Sens = 86.36%, Spec = 44.44%, PPV = 79.17%, NPV = 57.14%, Figure 3B) as well as in the whole patient collective (*p* = 0.004, Supplementary Figure 3). In contrast rs849142 (T allele) only correlated significantly with PFS in the training group (*p* = 0.05) (Supplementary Figure 4) but neither with OS in the training- nor PFS and OS in the validation group. rs849142 did not deviate significantly from Hardy-Weinberg equilibrium (*p* = 0.44, Supplementary Table 2). Also another SNP, rs463426, significantly correlated with ST in the validation- (*p* = 0.036) and whole group (*p* < 0.001), but showed only a trend for statistical significance in the training group (*p* = 0.066). Therefore, rs463426 was excluded from further analyses.

**Figure 4:**
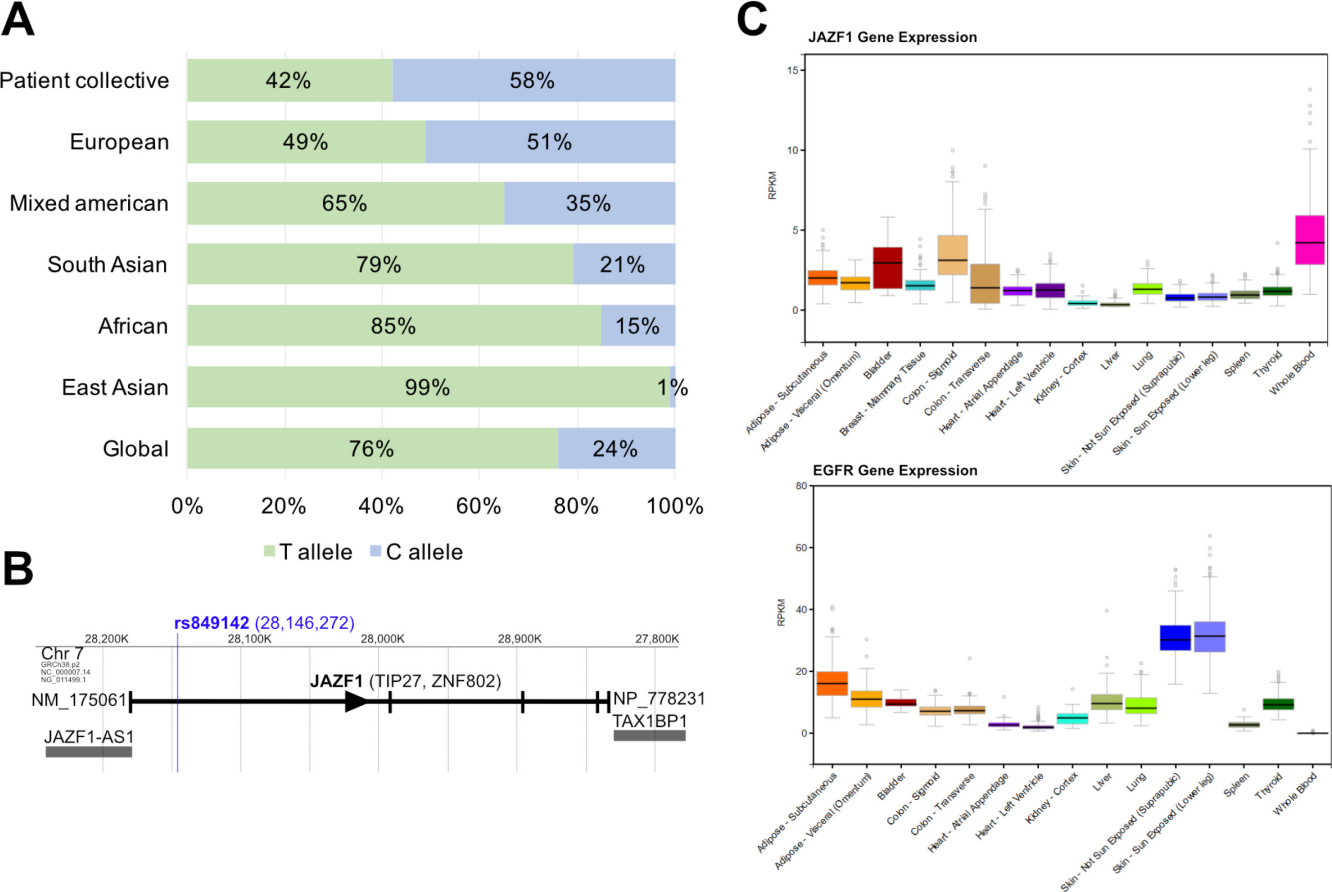
(**A**) Global- and sub-population allele frequencies of rs849142 (T>C) in comparison to the FIRE-3 patient collective as annotated at 1000genomes.org. (**B**) JAZF1 genomic region on Chromosome 7. *JAZF1* and adjacent genes are shown. Position of rs849142 is indicated by the blue line. Reading direction of *JAZF1* indicated by an arrowhead. Vertical bars indicate exons. *JAZF1*, Juxtaposed with another zinc finger protein 1. *JAZF1-AS1*, *JAZF1* antisense RNA 1. (**C**) mRNA expression of *JAZF1* (upper panel) and *EGFR* (lower panel) in various and ST related tissues as obtained from http://www.gtexportal.org. Expression is presented in RPKM (reads per kilobase of transcript per million mapped reads).

Global allele frequencies of rs849142 (T; C) are given as T: 0.765, C: 0.235, T|T: 0.615, C|C: 0.086, C|T: 0.299 (1000 Genomes Project) showing strong variations in different ethnicities (for details refer to Figure 4A). The European population displays almost balanced allele frequencies (T: 0.492, C: 0.508) compared to the East Asian population (T: 0.985, C: 0.015). Our results (Table 2) are expectedly in line with this data-set for the European subpopulation (T: 0.419, C: 0.581) which is another indicator for the support for the correctness of our measurements (Figure 4A). It turned out that the T-allele especially in the homozygous form (T|T) indicates high grade ST (Table 2).

Table 2Distribution of rs841942 in the patient collective

**Table:** 

Training	ST Grade 1	ST Grade 2	ST Grade 3	Sum
No variant call	5 (83.33%)	0 (0%)	1 (16.67%)	6
C/C	5 (100%)	0 (0%)	0 (0%)	5
C/T	1 (20%)	0 (0%)	4 (80%)	5
T/T	0 (0%)	0 (0%)	0 (0%)	0
Sum	11	0	5	16

**Table:** 

Validation	ST Grade 1	ST Grade 2	ST Grade 3	Sum
No variant call	1 (33.33%)	0 (0%)	2 (66.67%)	3
C/C	8 (57.14%)	3 (21.43%)	3 (21.43%)	14
C/T	8 (27.59%)	11 (37.93%)	10 (34.48%)	29
T/T	2 (22.22%)	2 (22.22%)	5 (55.56%)	9
Sum	19	16	20	55

### rs849142 is located in the JAZF1 gene

In a next step, we wanted to know if the genomic localization of rs849142 might help to elucidate a possible molecular mechanism. Therefore, database searches and *in silico* analyses were performed. rs849142 is located on chromosome 7 within an intronic region of the Juxtaposed with Another Zinc Finger protein 1 (*JAZF1*) gene (Figure 4B). JAZF1 is a transcriptional regulator that directly represses transcription or acts as a corepressor [26]. JAZF1 was not shown to be directly associated with ST but one of its interaction partners, Transforming growth factor β-activated kinase 1 (TAK1) is an integral component of signaling pathways involved in modulating the immune response [27]. The intronic position of rs849142 does not allow to conclude on a potential influence on transcriptional regulation of the *JAZF1* gene. Unfortunately, no skin samples from our patient collective or other ST studies were available to measure the *JAZF1* mRNA expression profile. It is known that *JAZF1* is ubiquitously expressed with highest levels in the adrenal gland and whole blood and lowest expression in the ST/ immune response-associated organs skin, spleen, and thyroid (expression data from: gtexportal.org (2)) (Figure 4C). Additionally, the expression of *JAZF1* correlates inversely with the levels of the cetuximab target EGFR in the skin and whole blood (Figure 4C). Taken together, these results suggest that *JAZF1* itself may not be directly involved in the development of cetuximab-mediated ST.

**Figure 5:**
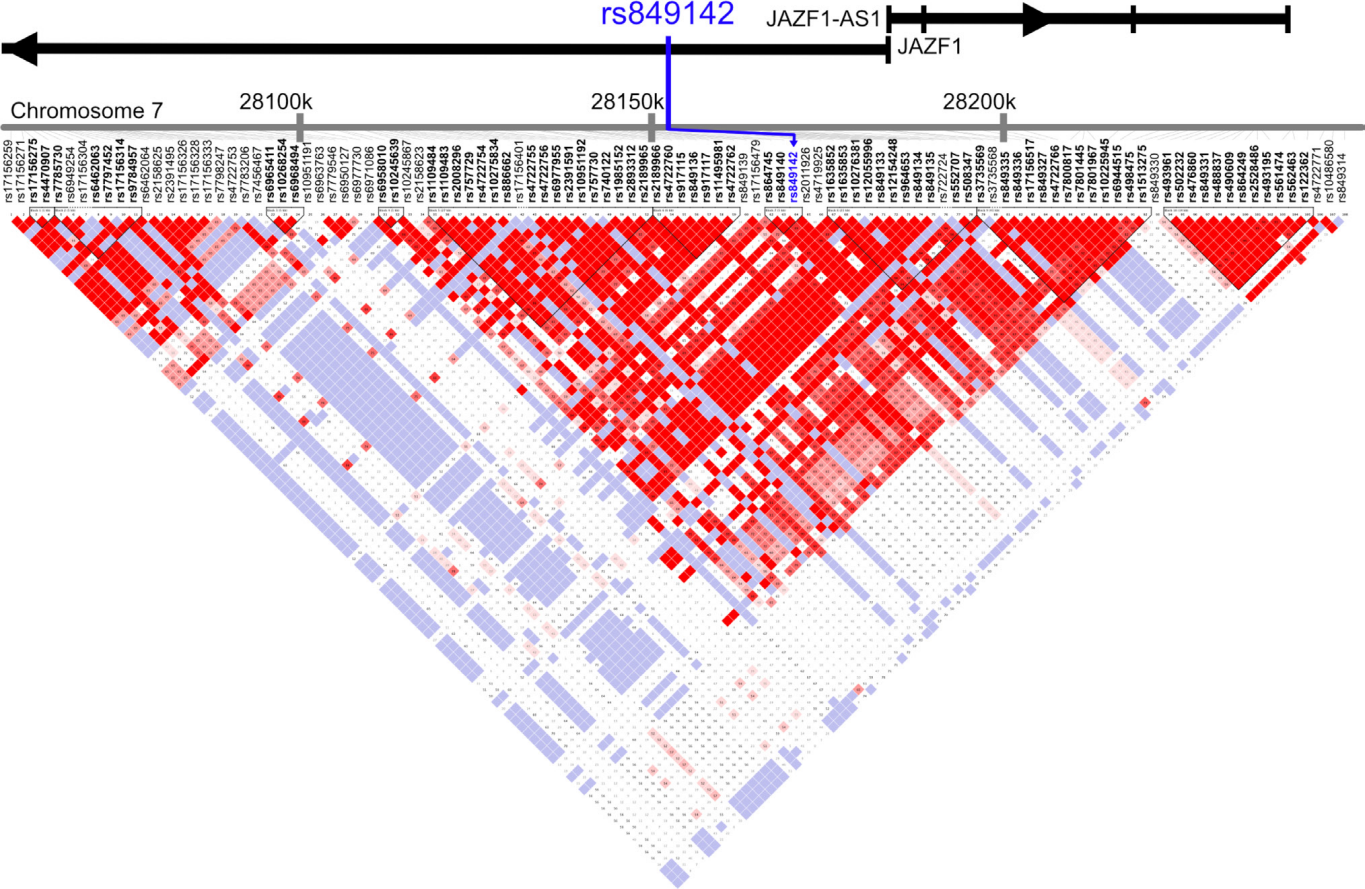
Linkage Disequilibrium (LD) Plot of Chromosome 7 28150kB ± 100 kB using Version 2 release 24 CEU (CEPH, Utah residents with ancestry from northern and western Europe) SNPs are presented in their genomic order. rs849142 is indicated by blue arrow. The reading direction (arrowhead) and exons (vertical bars) of the *JAZF1* and the partially overlapping *JAZF1-AS1* genes are indicated on top. The degree of positive correlation between two SNPs is visualized by color-coded squares at the intersection of orthogonal lines originating from each variant: White: low LD, low confidence; Shades of pink/red: low LD, high confidence; blue: high LD, low confidence; bright red: high LD, high confidence.

To explore a potential bystander effect indicated by linkage of rs849142 with other genes a linkage analysis of SNPs neighboring rs849142 was performed (Figure 5). rs849142 was only found in a coupling group with the SNPs rs864745 and rs849140 which are both located in the *JAZF1* gene. Therefore, rs849142 is also in linkage disequilibrium (LD) with several other SNPs within *JAZF1* and the adjacent non-protein coding *JAZF1-AS1* (antisense RNA 1) gene (Figure 4B). But also *JAZF1-AS1* has not been associated with ST, thus the connection of the SNP rs849142 and ST remains unknown like mutations in the *FOXL2* gene in granulosa cell tumors of the ovary [28]. Definitely, more research has to be invested.

## DISCUSSION

In our study collective, the SNP rs849142 (T allele) was identified and validated as a potential biomarker for skin-related adverse effects in mCRC patients under targeted anti-EGFR treatment using cetuximab (FIRE-3 study). Thus, genotyping of the rs849142 might help to improve decision-making on prophylactic ST treatment in the situation of targeted anti-EGFR treatment of left-sided and wildtypic (WT) KRAS- and NRAS gene mCRCs. In such a scenario, the high-risk group of patients with a T|T constellation of rs849142 might be considered for prophylactic ST treatment. As rs849142 is a genetic marker its testing has several advantages: First, higher sensitivity and specificity than measurements of variable day-to-day parameters like mRNA or serum components [13] and serum-level markers (AREG, EREG, HGF). Second, SNP detection can be measured easily from liquid biopsies (blood, saliva) and is less invasive than taking tissue biopsies. Third, SNP-typing can be performed together with the testing for mutations in the KRAS- und NRAS-genes as the predictive test for using anti-EGFR targeting antibodies in the therapy of mCRC. Due to the different global distribution of rs849142 alleles in distinct ethnicities SNP-testing might be especially valuable for Europeans (T: 49.2%, C: 50.8%) and Americans (T: 65%, C: 35%) but less for Africans (T: 84.9%, C:15.1%) and especially East Asians (T: 98.5%, C:1.5%). Due to the small size of our collections, the missing correlation with survival as well as underling molecular mechanism, these results are preliminary. Further research especially validation in larger cohorts is essential.

For our aim to find biomarkers predicting ST in anti-EGFR treatment of mCRC patients, we chose a training/ validation approach in a patient collective of the FIRE-3 study [17, 18]. Our kind of approach includes a high bias as the statistic variance is high due to the low amounts of events. For this reason seldom events might be missed. The alternative is a linkage analysis which requires numbers of events in the range of several hundreds to thousands. It is questionable if such high numbers of patients with documented ST grade are accessible even globally. Another major drawback of small collections might be a drift in the representativity compared to the whole collection. Therefore, we confirmed by applying an additional ST unrelated parameter, namely the response to anti-EGFR directed therapy [9], that our sub-selection still reflected the whole FIRE-3 patient cohort. Another bias in training/ validation approaches is the need for an input of pre-selected parameters; in our case the collection of SNPs under investigation. Therefore, three non-oncological diseases were chosen, because ST in mCRC patients is most likely a tumor independent effect. 1: Acne is a common disease linked to deregulation and changes in the skin environment [29]. 2: SLE is an autoimmune disease that commonly results in skin-related manifestations [30]. 3: Imunoglobulin fragment crystallizable receptors (FcR) are responsible for unspecific binding of antibody-classes via the Fc-region of the antibody which might result in the joining of tumor- with other (immune) cells eventually resulting in antibody-dependent cell-mediated cytotoxicity (ADCC) or other kinds of interference. SNPs are known to influence the affinity of FcRs for immunoglobulins [31]. As cetuximab is an IgG1 antibody the class of FcRγ was of interest for our study. This approach resulted in a total of 61 SNPs which are known to modulate acne, SLE, and FcRγ binding. Of these only rs849142—found in the SLE group—was significantly associated with ST in both the training and validation group.

As ST correlates with survival of mCRC patients [9], it was reasonable to check if this was also true for rs849142. Unexpectedly, a significant association of rs849142 (T allele) was only observed for PFS in the training group. No correlation was present with OS in the training set nor PFS and OS in the validation group. Thus, ST and response or survival seem to share several but differ in other characteristics. Underlying molecular mechanisms are unknown and thus arguing would be speculative. Moreover, as rs849142 is located in the intron of *JAZF1* aside from functional relevant sites like splice donor/acceptor it is idle to discuss a possible role or function. For sure both open questions need further research.

Taken together, our study revealed rs849142 as a potentially predictive biomarker of ST in cetuximab treated mCRC patients. Patients may largely benefit from a prediction of ST and potentially the severity of the disease by genotyping rs849142 as physicians could manage ST in the relevant population of patients thereby treating only that subset of patients where ST will occur as an AE of the anti-EGFR targeted treatment. Moreover, reading out the genotype of the rs849142 alleles can easily be incorporated into the detection pipeline for the analysis of other relevant molecular biomarkers for mCRC, namely mutations in the BRAF-, KRAS- and NRAS-genes as well as microsatellite instability [32]. Finally, it is important to note that our finding has to be validated in a larger cohort or at best in the setting of a prospective clinical study.

## MATERIALS AND METHODS

### Patients

The FIRE-3 study was a randomized phase 3 study of stage IV mCRC patients (age 18–75 years) treated with the combinatorial-chemotherapy (CTX) folinic acid, leucovorin, 5-Fluoruracil (5-FU), and Irinotecan (FOLFIRI) with either cetuximab or bevacizumab. The inclusion criteria for FIRE-3 study have been described in detail before [17]. Further inclusion criteria for this particular analysis were: Appropriate follow-up data, cetuximab treatment group presence of remaining DNA samples. ST grades were determined using the common toxicity criteria of adverse events (CTCAE) version 4.0. For a training-validation approach, two subgroups of the cetuximab treatment arm were analyzed: 1) training group (*n* = 16) consisting of patients with grade one and three ST; 2) validation group (*n* = 55) including patients with ST grade one, two, and three. For this approach, from all eligible patients showing ST grade 1 or 3, sixteen were assigned randomly to the training group. All other eligible patients were included into the validation group before the analysis.

### DNA extraction and genotyping

Genomic DNA was isolated from peripheral blood mononuclear cells using QIAGEN blood kits following the vendor's recommendations and used as the template for a targeted next-generation-sequencing (NGS) approach together with custom sequencing panels. Therefore, three Ion Ampliseq™ Custom DNA-Hotspot panels were designed utilizing Ion Torrent Ampliseq^®^ Custom Panel Designer (Supplementary Table 1). They were used together with Ampliseq™ Library kits for preparing libraries which were analyzed on an NGS IonTorrent PGM™ (Personal Genome Machine, Thermo Fisher Scientific) platform. The experimental procedures were done according to the manufacturer's manual. Briefly, DNA concentration and quality was measured using the Qubit™ 3 Fluorimeter (Thermo Fisher Scientific). The concentration of amplifiable DNA was quantified using an RNase P-gene specific detection system (Roche Diagnostics). Samples with a ratio of >2 were extracted again. Amplicon libraries were created using the Ion Ampliseq™ Library Kit 2.0 (Thermo Fisher Scientific) with 21 PCR cycles. For comparable coverage of the samples, DNA concentration was controlled for by the Ion Torrent qPCR quantification following the vendor's instructions. Groups of 16 libraries were transferred into the IonChef™ pipetting station for clonal amplification by emulsion PCR and Ion-316™ Chip loading. Chips were loaded onto the IonTorrent PGM™ (Personal Genome Machine, Thermo Fisher Scientific).

### Statistical analysis

Analysis of data obtained by NGS was done applying the Ion Reporter™ v.4.6 software: data quality was controlled for by application of filter criteria recommended by the vendor. Reads were mapped to the reference genome hg19 and variants were called using the standard settings. SNP annotation was based on dbSNP138. Resulting variant calling files (VCF) were reformatted to match specifications of the PLINK software version 1.07 [25]. Of note, SNPs showing more than two alleles were excluded from the analysis, because they cannot be evaluated in a typical quantitative genetic association analysis. Overview tables on quality based on patients and variants were calculated using the summary statistics function of the PLINK software. The fraction of successfully genotyped variants was visualized in Haploview version 4.2 (Supplementary Figures 3 and 4) [33]. The exact Hardy-Weinberg equilibrium (HWE) test module of PLINK was used for calculating deviations from HWE applying a significance level of *p* < 0.001. Genotype-phenotype-association was estimated for each group using the Wald statistic in PLINK measuring ST as a quantitative marker. Results were visualized as Manhattan plots (Haploview software). Sensitivity [Sens], specificity [Spec], positive predictive value [PPV] and negative predictive value [NPV] were calculated comparing the genotype corresponding to ST grade 1 and to all other genotypes and ST grades. Linkage disequilibrium analysis of a region spanning 100 kb upstream and downstream of rs849142 was run with the Haploview software and haplotype blocks were calculated [34]. Pooled over strata *p*-values of survival analyses were calculated with the SPSS 23.0 software using log-rank- and Kaplan–Meier models.

## SUPPLEMENTARY MATERIALS FIGURES AND TABLES



## Abbreviations

ADCC: antibody-dependent cell-mediated cytotoxicity
AE: adverse effect
CTCAE: Common Toxicity Criteria for Adverse Events
CTX: combinatorial-chemotherapy
EGFR: epidermal growth factor receptor
FcR: Imunoglobulin fragment crystallizable receptors
FOLFIRI: chemotherapy scheme folinic acid: leucovorin: 5-Fluoruracil (5-FU): and Irinotecan
HWE: Hardy-Weinberg equilibrium
JAZF1: juxtaposed with another zinc finger protein 1
mCRC: metastatic colorectal cancer
NGS: Next-Generation-Sequencing
NPV: negative predictive value
OS: overall survival
PFS: progression free survival
PPV: positive predictive value
Sens: sensitivity
SLE: systemic lupus erythematosus
SNP: single nucleotide polymorphism
Spec: specificity
ST: skin toxicity.
