# Supplementary material for: The DNA-polymorphism rs849142 is associated with skin toxicity induced by targeted anti-EGFR therapy using cetuximab
Source: Oncotarget. 2018 Jul 13;9(54):30279–88. doi: 10.18632/oncotarget.25689 (PMC6084390; doi:10.18632/oncotarget.25689)
Supplement: Supplementary file 1 [file oncotarget-09-30279-s001.pdf]

The DNA-polymorphism rs849142 is associated with skin toxicity induced by targeted anti-EGFR therapy using cetuximab

SUPPLEMENTARY MATERIALS

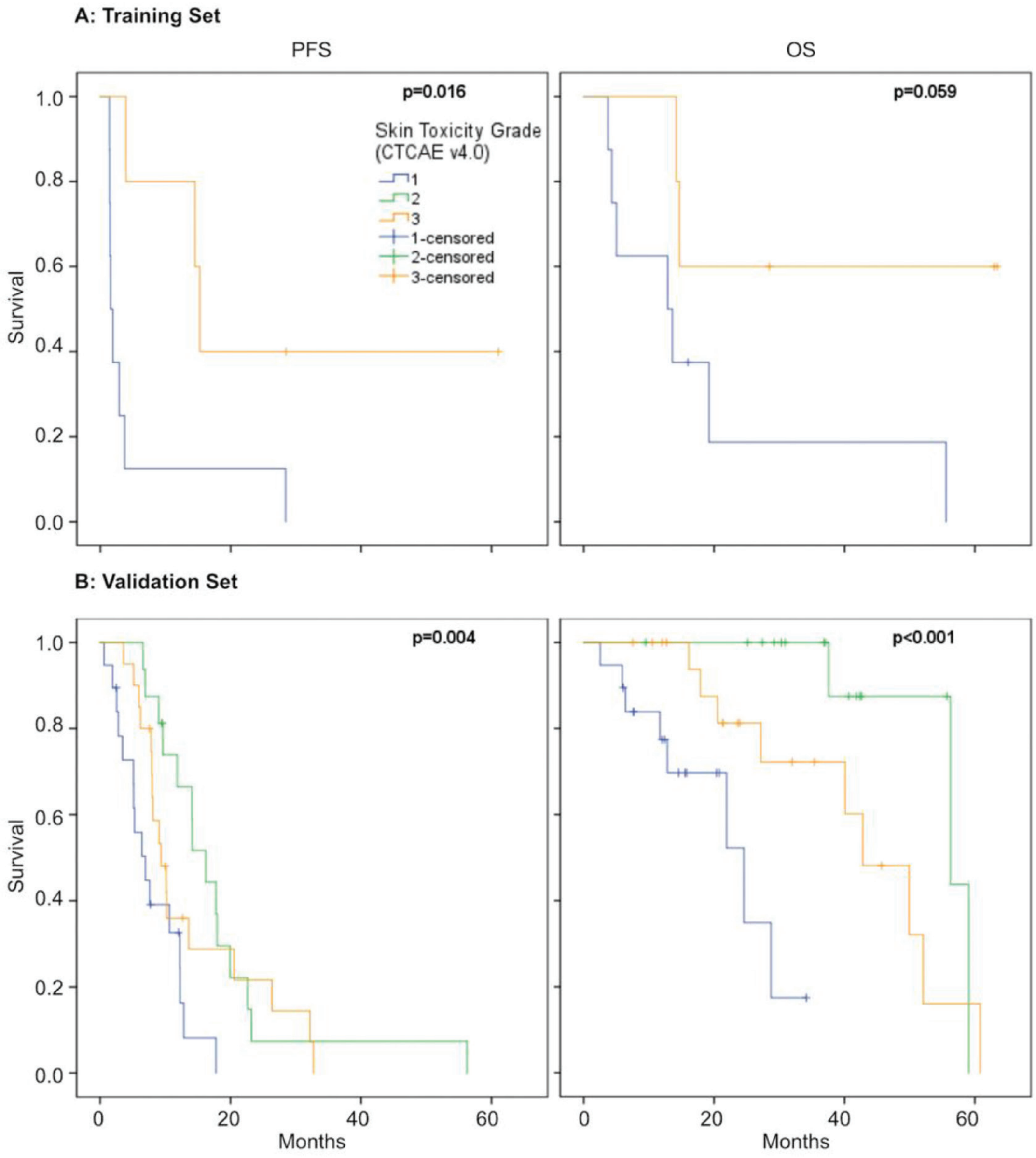

Supplementary Figure 1: PFS and OS Kaplan-Meier plots for cetuximab associated ST grades in the Training (**A**) and Validation (**B**) groups.

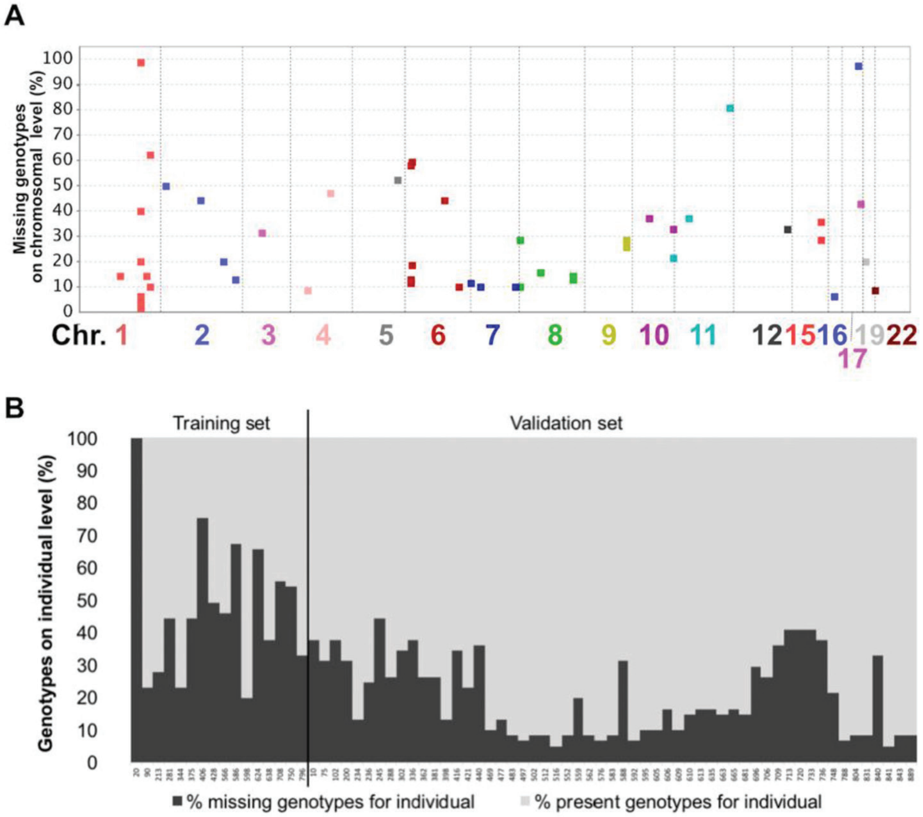

Supplementary Figure 2: (**A**) Proportion of missing genotypes for each SNP. SNP position on the chromosome is indicated by the horizontal position. (**B**) Sequencing quality in the patient collective.

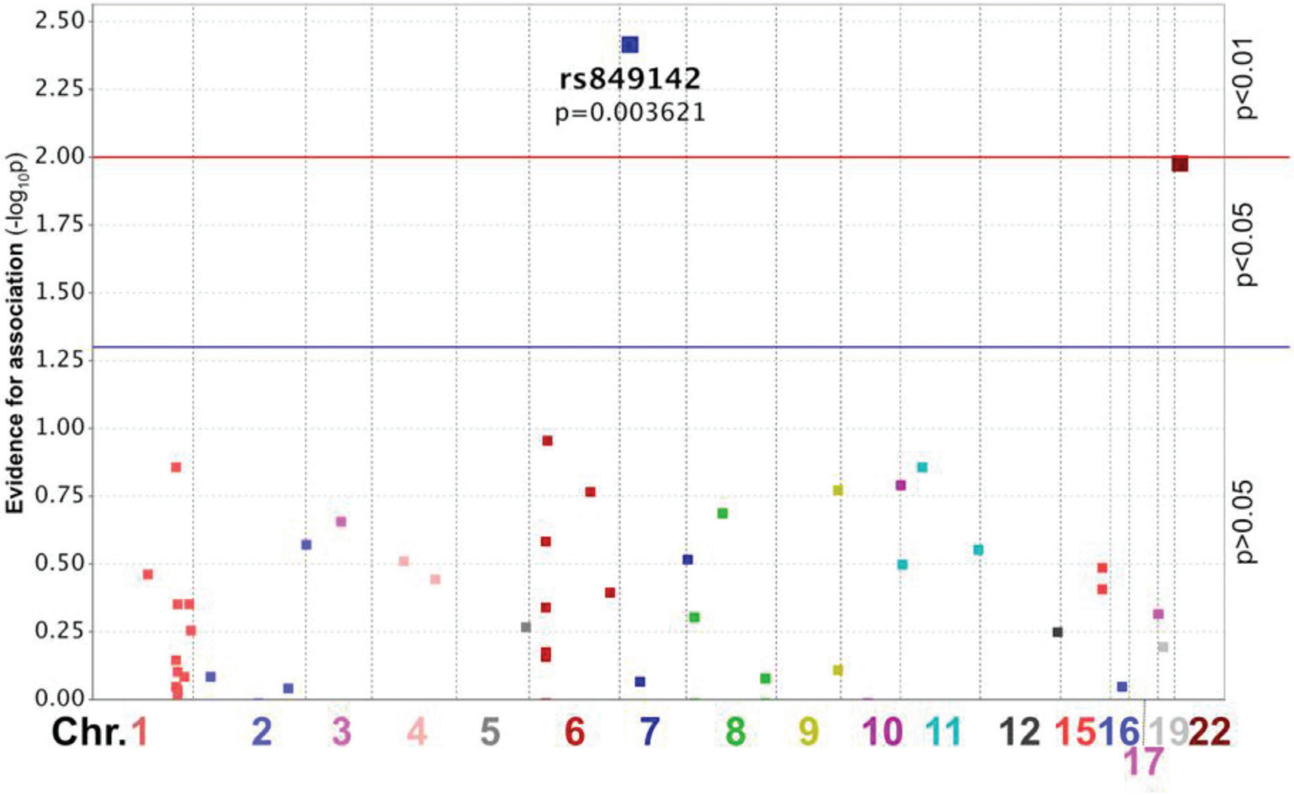

Supplementary Figure 3: Manhattan plot for ST of all patients (Training and Validation group combined). SNP position on the chromosome is indicated by horizontal position. Y-axis value describes magnitude of evidence for association calculated as $-\log_{10}$ (*p* value). Chr., chromosome.

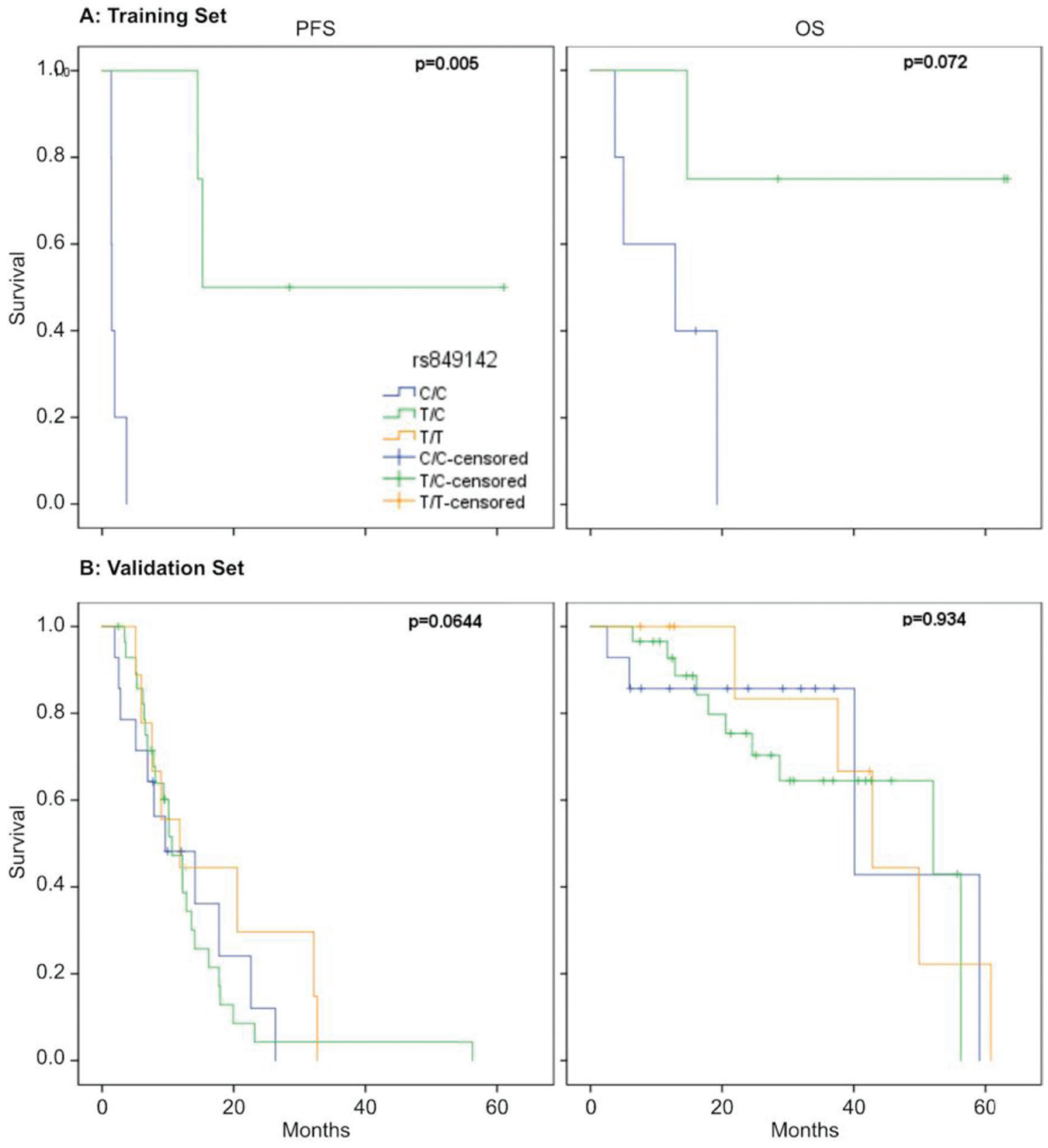

Supplementary Figure 4: Kaplan–Meier analysis of the association of PFS or OS with the rs849142 genotype in the Training (**A**) and Validation group (**B**). Heterozygous and homozygous combinations of the C and T alleles were analyzed.

Supplementary Table 1: Overview of sequencing panels

- **Acne sequencing panel**

Chr	Start	End	SNP	REF	Panel
chr4	154626316	154626317	rs5743708	REF=G;OBS=A;ANCHOR=C	acne
chr9	120475301	120475302	rs4986790	REF=A;OBS=G;ANCHOR=G	acne
chr9	120475601	120475602	rs4986791	REF=C;OBS=T;ANCHOR=T	acne
chr6	31543100	31543101	rs361525	REF=G;OBS=A;ANCHOR=T	acne
chr6	31543030	31543031	rs1800629	REF=G;OBS=A;ANCHOR=T	acne
chr6	31542481	31542482	rs1799724	REF=C;OBS=T;ANCHOR=T	acne
chr6	31542475	31542476	rs1800630	REF=C;OBS=A;ANCHOR=C	acne
chr6	31542307	31542308	rs1799964	REF=T;OBS=C;ANCHOR=T	acne
chr2	113537222	113537223	rs17561	REF=C;OBS=A;ANCHOR=T	acne
chr15	75012984	75012985	rs1048943	REF=T;OBS=C;ANCHOR=T	acne
chr15	75011640	75011641	rs4646903	REF=A;OBS=G;ANCHOR=C	acne
chr10	104597151	104597152	rs743572	REF=A;OBS=G;ANCHOR=T	acne
chr8	128676130	128676131	rs4133274	REF=A;OBS=G;ANCHOR=T	acne
chr8	128691211	128691212	rs13248513	REF=T;OBS=C;ANCHOR=T	acne

- **SLE sequencing panel**

Chr	Start	End	SNP	REF	Panel
chr16	31313252	31313253	rs9888739	REF=C;OBS=T;ANCHOR=T	sle
chr6	106568033	106568034	rs548234	REF=C;OBS=T;ANCHOR=T	sle
chr6	138196065	138196066	rs2230926	REF=T;OBS=G;ANCHOR=T	sle
chr5	150457484	150457485	rs7708392	REF=G;OBS=C;ANCHOR=T	sle
chr22	21809184	21809185	rs463426	REF=T;OBS=C;ANCHOR=T	sle
chr11	128311058	128311059	rs6590330	REF=G;OBS=A;ANCHOR=T	sle
chr7	50305862	50305863	rs4917014	REF=T;OBS=G;ANCHOR=T	sle
chr11	35129171	35129172	rs507230	REF=G;OBS=A;ANCHOR=T	sle
chr4	102751075	102751076	rs10516487	REF=G;OBS=A;ANCHOR=T	sle
chr8	11340180	11340181	rs7812879	REF=T;OBS=C;ANCHOR=T	sle
chr8	56849385	56849386	rs7829816	REF=A;OBS=G;ANCHOR=T	sle
chr2	33701889	33701890	rs13385731	REF=T;OBS=C;ANCHOR=T	sle
chr1	183549756	183549757	rs10911363	REF=G;OBS=T;ANCHOR=T	sle
chr2	191964632	191964633	rs7574865	REF=T;OBS=G;ANCHOR=T	sle
chr1	114377567	114377568	rs2476601	REF=A;OBS=G;ANCHOR=T	sle
chr1	173191474	173191475	rs2205960	REF=G;OBS=T;ANCHOR=T	sle
chr6	32408496	32408497	rs3135394	REF=A;OBS=G;ANCHOR=T	sle
chr12	129299384	129299385	rs10847697	REF=G;OBS=A;ANCHOR=T	sle
chr7	128588999	128589000	rs2070197	REF=T;OBS=C;ANCHOR=T	sle
chr11	589563	589564	rs4963128	REF=T;OBS=C;ANCHOR=T	sle
chr16	86018632	86018633	rs2280381	REF=C;OBS=T;ANCHOR=T	sle
chr2	163124050	163124051	rs1990760	REF=C;OBS=T;ANCHOR=T	sle
chr19	10472932	10472933	rs280519	REF=A;OBS=G;ANCHOR=T	sle
chr10	50119053	50119054	rs1913517	REF=A;OBS=G;ANCHOR=T	sle
chr3	58370176	58370177	rs6445975	REF=G;OBS=T;ANCHOR=T	sle
chr7	28185890	28185891	rs849142	REF=T;OBS=C;ANCHOR=T	sle

chr6	34824635	34824636	rs11755393	REF=A;OBS=G;ANCHOR=T	sle
chr8	10761584	10761585	rs6985109	REF=G;OBS=A;ANCHOR=T	sle
chr1	161514595	161514596	rs396716	REF=A;OBS=G;ANCHOR=T	sle
chr1	161514541	161514542	rs396991	REF=A;OBS=C;ANCHOR=T	sle
chr1	161512347	161512348	rs445509	REF=C;OBS=T;ANCHOR=C	sle
chr1	161512958	161512959	rs1042206	REF=A;OBS=C;ANCHOR=A	sle
chr1	161518213	161518214	rs148181339	REF=T;OBS=C;ANCHOR=A	sle
chr1	161643797	161643798	rs1050501	REF=T;OBS=C;ANCHOR=A	sle
chr17	4637885	4637886	rs1051009	REF=G;OBS=A;ANCHOR=T	sle
chr1	183086237	183086238	rs10752900	REF=G;OBS=A;ANCHOR=T	sle

- **FcGγ sequencing panel**

Chr	Start	End	SNP	REF	Panel
chr1	161518332	161518333	rs10127939	REF=A;OBS=C;ANCHOR=G	FCGR
chr1	161518233	161518234	rs145557772	REF=G;OBS=A;ANCHOR=C	FCGR
chr1	161514541	161514542	rs396991	REF=A;OBS=C;ANCHOR=A	FCGR
chr1	161514595	161514596	rs396716	REF=A;OBS=G;ANCHOR=T	FCGR
chr1	161514627	161514628	rs443082	REF=C;OBS=T;ANCHOR=G	FCGR
chr1	161512347	161512348	rs445509	REF=C;OBS=T;ANCHOR=C	FCGR
chr1	161512958	161512959	rs1042206	REF=A;OBS=C;ANCHOR=A	FCGR
chr1	161518213	161518214	rs148181339	REF=T;OBS=C;ANCHOR=A	FCGR
chr1	161479744	161479745	rs1801274	REF=A;OBS=G;ANCHOR=C	FCGR
chr1	161548496	161548497	rs428888	REF=T;OBS=C;ANCHOR=T	FCGR
chr1	161548632	161548633	rs403016	REF=C;OBS=G;ANCHOR=C	FCGR
chr1	161643797	161643798	rs1050501	REF=T;OBS=C;ANCHOR=A	FCGR
chr1	161484563	161484564	rs6427598	REF=T;OBS=A;ANCHOR=G	FCGR
chr1	161484209	161484210	rs368433	REF=T;OBS=C;ANCHOR=A	FCGR
chr1	161581052	161581058	rs3219018	REF=G;OBS=C;ANCHOR=T	FCGR
chr1	161647532	161647533	rs844	REF=A;OBS=G;ANCHOR=T	FCGR
chr1	161645470	161645471	rs1674761	REF=A;OBS=C;ANCHOR=T	FCGR
chr1	161641295	161641296	rs5017567	REF=A;OBS=C;ANCHOR=C	FCGR
chr1	161646823	161646824	rs12118043	REF=C;OBS=A;ANCHOR=C	FCGR
chr1	161599653	161599654	rs5030738	REF=G;OBS=T;ANCHOR=A	FCGR

Supplementary Table 2: Hardy-Weinberg equilibrium analysis

Chr	SNP	p Hardy-Weinberg
1	rs2476601	0.3953
1	rs1801274	0.2965
1	rs368433	1.0000
1	rs6427598	0.3298
1	rs445509	1.0000
1	rs1042206	1.0000
1	rs396991	0.1769
1	rs396716	1.0000
1	rs443082	1.0000
1	rs148181339	1.0000
1	rs145557772	1.0000
1	rs10127939	1.0000
1	rs5030738	1.0000
1	rs5017567	1.0000
1	rs1050501	1.0000
1	rs1674761	1,51E-08
1	rs12118043	1.0000
1	rs844	1.0000
1	rs2205960	1.0000
1	rs10752900	0.8005
1	rs10911363	0.1303
2	rs13385731	1.0000
2	rs17561	0.6911
2	rs1990760	0.5525
2	rs7574865	0.5010
3	rs6445975	1.0000
4	rs10516487	0.5785
4	rs5743708	1.0000
5	rs7708392	1.0000
6	rs1799964	1.0000
6	rs1800630/rs4645836	0.5806
6	rs1799724	1.0000
6	rs1800629	1.0000
6	rs361525	1.0000
6	rs3135394	1.0000
6	rs11755393	0.7853
6	rs548234	0.3124
6	rs2230926	1.0000
7	rs849142	0.4356
7	rs4917014	0.5478
7	rs2070197	1.0000
8	rs6985109	0.5629
8	rs7812879	0.5965

8	rs7829816	1.0000
8	rs4133274	1.0000
8	rs13248513	1.0000
9	rs4986790	1.0000
9	rs4986791	1.0000
10	rs1913517	0.7621
10	rs743572	1.0000
11	rs4963128	0.0413
11	rs507230	1.0000
11	rs6590330	0.2092
12	rs10847697	0.09936
15	rs79812015/rs4646903	1.0000
15	rs1048943	1.0000
16	rs9888739	0.6566
16	rs2280381	1.0000
17	rs1051009	0.3535
19	rs280519	0.05934
22	rs463426	1.0000
